# Association between troponin and NT-proBNP levels, cytokines, and clinical outcomes in early sepsis response: a cohort study

**DOI:** 10.1016/j.bjane.2025.844712

**Published:** 2025-11-15

**Authors:** Wagner Nedel, Luis Valmor Portela

**Affiliations:** a Grupo Hospitalar Conceição, Unidade de Terapia Intensiva, Porto Alegre, RS, Brazil; b Universidade Federal do Rio Grande do Sul, Programa de Pós-Graduação em Ciências Pneumológicas, Porto Alegre, RS, Brazil; c Universidade Federal do Rio Grande do Sul, Instituto de Ciências Básicas da Saúde, Laboratório de Neurotrauma e Biomarcadores, Porto Alegre, RS, Brazil

Dear Editor,

N-Terminal pro-B-type Natriuretic Peptide (NT-proBNP) and troponin are well-established biomarkers indicative of the severity of cardiac injury. An increase in preload stimulates ventricular myocytes to synthesize and release NT-proBNP. Troponin is frequently evaluated in critically ill patients with sepsis, with its release attributed to various mechanisms, including supply-demand mismatch and direct myocardial inflammation. Additionally, both biomarkers exhibit impaired renal clearance. They are associated with cardiomyocyte stress and myocardial inflammatory response and may be elevated in various inflammatory conditions affecting the heart.^1^ The dysregulated inflammatory response to infection in sepsis often leads to unfavorable clinical outcomes.^2^ Accordingly, elevated NT-proBNP and troponin levels have been correlated with worse outcomes, as well as with increased Sequential Organ Failure Assessment (SOFA) scores, reflecting more severe multiorgan injury and unresolved inflammation. Interleukin (IL)-6, IL-1β, IL-10, and C-Reactive Protein (CRP) are important markers of an early dysregulated inflammatory response in sepsis and are prognostic biomarkers of poor outcomes.^3^^,^^4^ Additionally, the release of IL-1β and IL-6 during endotoxemia can lead to myocardial depression, suggesting that they may lead to myocardial damage.^5^ Nonetheless, the relationship between the early elevation of interleukins and the levels of serum troponin and NT-proBNP has not been sufficiently investigated. Therefore, the main objective of this study is to evaluate the association between myocardial injury markers and pro- and anti-inflammatory ILs in the initial response of sepsis, as well as the association between these levels and early improvement in sepsis response.

We performed a post-hoc study derived from a cohort study that prospectively evaluated consecutive patients who had been admitted to four different ICUs (Grupo Hospitalar Conceição, Porto Alegre, Brazil). The original cohort was described previously.^6^ This study was approved by the local ethics committee (Plataforma Brazil number 66240017.0.0000.5530).

We included adult patients admitted to the ICU with sepsis and persistent hypotension. Sepsis and persistent hypotension were defined according to the current guidelines.^7^ Patients were excluded if they presented with known mitochondrial disease, pregnancy, refusal of the patient or next of kin to sign the informed consent, imminent death, withholding or withdrawing treatment, or acute coronary syndrome concomitant with a sepsis diagnosis.

The epidemiological characteristics were prospectively recorded, including the Simplified Acute Physiology Score (SAPSIII), SOFA score at admission to the ICU, and SOFA score on day-3. We measured NT-proBNP, troponin, IL-1β, IL-6, IL-10, and C-reactive protein (CRP) levels upon the diagnosis of sepsis. The primary objectives are to assess the correlation between troponin and NT-proBNP levels with ILs. The secondary objectives involve evaluating the correlation between NT-proBNP and troponin levels and clinical, hemodynamic, and CRP levels. Clinical variables are presented in Table 1.

**Table 1:** Clinical and laboratory variables associated with 28 days mortality.

Variable	Survivors (n = 48) Mean/median (sd/iqr) or ratio	Non-survivors (n = 42) Mean/median (sd/iqr) or ratio	MD (95% CI), Median difference (95% CI), OR (95% CI), p-value
Age	64.1 (15.5)	67 (16.6)	MD -2 (-8 to 4), p = 0.64
SAPS3	73 (12)	79 (12.8)	MD -7 (-12 to -2), p = 0.03
CRT	4 (2 – 4)	4.5 (3 – 7)	Median Difference -1 (-2 to -1), p = 0.02
Cumulative fluid balance day-1	2650 (1151 – 4469)	4375 (2025 – 5857)	Median Difference -1241 (-2466 to -35), p = 0.04
NE maximum day-1	0.18 (0.1 – 0.3)	0.35 (0.19 – 0.61)	Median Difference -0.15 (-0.27 to -0.04), p < 0.01
Mottling score day-1	0 (0 – 0)	0 (0 – 2)	Median Difference 0 (0 to 0), p < 0.01
SOFA	7 (6 – 9)	9 (7 – 11)	Median Difference -2 (-3 to -1), p ≤ 0.01
IL-6	63 (30.2 – 195.2)	122.9 (32.7 – 217)	Median Difference -9 (-94 to 25), p = 0.58
IL-10	174.9 (125.8 – 224.9)	200 (168.4 – 246.7)	Median Difference -30 (-76 to 6), p = 0.1
IL-1β	23.6 (13.9 – 458.4)	56.3 (17.8 – 738.7)	Median Difference -23 (-163 to 4), p = 0.14
CRP	156 (79 – 212)	206 (112 – 290)	Median Difference -50 (-101 to -1), p = 0.04
Hemodialysis	6/48	23/42	OR 8.47 (2.96 – 24.2), p < 0.01
MV	37/48	40/42	OR 5.94 (1.23 – 57.65), p = 0.01
Male sex	30/48	20/42	OR 0.54 (0.23 – 1.26), p = 0.2
Malignancy	10/48	9/42	OR 1.03 (0.37 – 2.85), p = 1
COPD	7/48	5/42	OR 0.79 (0.23 – 2.71), p = 0.76
Diabetes	15/48	10/42	OR 0.68 (0.27 – 1.75), p = 0.48
Hypertension	17/48	15/42	OR 1.01 (0.42 – 2.4), p = 1
Ischemic cardiomiopathy	10/48	7/42	OR 0.76 (0.26 – 2.21), p = 0.78
Heart failure	11/48	5/42	OR 0.45 (0.14 – 1.43), p = 0.27
Troponin	58 (25 – 120)	71 (36 – 138)	Median difference 12 (-15 to 40), p = 0.36
NT-proBNP	2413 (976 – 8169)	4409 (1851 – 17750)	Median difference 1079 (-717 to 3621), p = 0.28

COPD, Chronic Obstructive Pulmonary Disease; CRP, C-Reactive Protein; CRT, Capillary Refill Time; IL, Interleukin; MD, Mean Difference; MV, Mechanical Ventilation; NE, Norepinephrine; NT-proBNP, N-Terminal pro-B-type Natriuretic Peptide; OR, Odds Ratio; SAPS, Simplified Acute Physiology Score; SOFA, Sequential Organ Failure Assessment.

Patients with ischemic cardiomyopathy (n = 16) did not have significantly increased troponin levels as compared to those who did not have ischemic cardiomyopathy (n = 60): 75 ng.L^-1^ (56–360) vs. 59 ng.L^-1^ (28–114), p = 0.18; and NT-proBNP levels: 3143 pg.mL^-1^ (1003–10369) vs. 3503 pg.mL^-1^ (2224–10643), p = 0.49. In addition, patients with heart failure (n = 15) did not have significantly increased troponin levels as compared to those who did not have heart failure (n = 61): 119 ng.L^-1^ (47–280) vs. 62 ng.L^-1^ (33–111), p = 0.10; as well as NT-proBNP levels: 7390 pg.mL^-1^ (1719–19865) vs. 3455 pg.mL^-1^ (1004–7732), p = 0.24.

Seventy-eight patients had CRP measurements, and 64 patients had IL-6, IL-10 and IL-1β measurements. Troponin levels were not associated with interleukin or CRP levels at the same time. The Pearson’s coefficients were -0.02 (95% CI -0.24 to 0.29, p = 0.84) for IL-6; 0.01 (95% CI -0.25 to 0.28, p = 0.90) for IL-10; -0.14 (95% CI -0.39 to 0.13, p = 0.30) for IL-1β; and -0.02 (95% CI -0.25 to 0.21, p = 0.87) for CRP. Remarkably, there was an association between NT-proBNP and IL-6 levels, with a Pearson’s coefficient of 0.3 (95% CI 0.03 to 0.52, p = 0.03), as well as between NT-proBNP levels and IL-10 levels, with a Pearson’s coefficient of 0.34 (95% CI 0.07 to 0.56, p = 0.01). Furthermore, NT-proBNP levels were not associated with IL-1β levels (Pearson’s coefficient 0.04 [95% CI -0.23 to 0.30]; p = 0.78) or CRP levels (Pearson’s coefficient 0.02 [95% CI -0.21 to 0.26]; p = 0.83). In an analysis using a general linear model, we found an interaction between NT-proBNP levels and IL-6 levels (β-coefficient 0.34 [95% CI 0.08–0.6], p = 0.01) and IL-10 levels (beta-coefficient 0.37 [95% CI 0.11–0.62], p < 0.01). This interaction was independent of heart failure or ischemic cardiomyopathy status. We analyzed the maximum Norepinephrine (NE) dose as a marker of hemodynamic dysfunction during sepsis. Troponin was not associated with the maximum NE dose: Pearson’s coefficient -0.01 (95% CI -0.24 to 0.21, p = 0.88); but NT-proBNP was: Pearson’s 0.3 (95% CI 0.07 to 0.49, p = 0.01).

We conducted a multivariate analysis of troponin and NT-proBNP and their association with 28-day mortality, adjusted for potential confounders: diagnosis of ischemic cardiomyopathy, diagnosis of heart failure, the cumulative fluid balance at day-1, the maximum NE dose at day-1, SAPS3 score, SOFA score at day-1, sex and CRP levels at day-1. In this modeling, troponin (OR = 1.0, 95% CI 0.99–1.0) and NT-proBNP (OR = 1.0, 95% CI 0.98–1.0) were not associated with the outcome.

Patients with an improved SOFA score (n = 51) on day-3 had lower troponin levels than those who did not improve SOFA score on day-3 (n = 25): 49 ng.L^-1^ (22–85) vs. 113 ng.L^-1^ (72–334), respectively; Mean Difference (MD) 53 ng.L^-1^ (95% CI 19–90), p < 0.01. Patients who improved their SOFA score (n = 50) on day-3 did not display lower NT-proBNP levels than those who did not improve their SOFA score on day-3 (n = 23): 2861 pg.mL^-1^ (1036–7532) vs. 5834 pg.mL^-1^ (1509–21163), MD = 1256 pg.mL^-1^ (95% CI -724 to 5441), p = 0.26.

Early response in patients with sepsis shows increased troponin and NT-proBNP levels, and our data reiterate these findings.^8^ Our results indicate distinct responses of troponin and NT-proBNP in the early stages of sepsis. Higher NT-proBNP levels were correlated with increased IL-6 and IL-10 expression and more severe hemodynamic instability during the acute phase, suggesting a similar acute profile between inflammatory and cardiac biomarkers. Because this kind of interaction, these associations may merely represent different indicators of patient severity. Nonetheless, troponin was associated with an improvement in the SOFA score, an important marker of clinical improvement in sepsis.^9^ Elevated troponin and NT-proBNP levels in critically ill non-cardiac patients are associated with disease severity,^10^ but were not associated with mortality. The small sample size limits definitive conclusions regarding these associations. Also, we performed multiple analyses of several variables and outcomes addressed in the study, but we did not correct the p-value for multiple interactions. Our study was designed to be hypotheses-generating, and our findings necessitate validation through adequately powered, prospectively designed studies that incorporate serial biomarker sampling and account for potential confounding variables that may affect cardiac biomarker measurements.

## Data availability statement

The datasets generated and/or analyzed during the current study are available from the corresponding author upon reasonable request.

## AI assistant disclosure

The authors used Paperpal to polish the language. The authors reviewed all suggested changes, and the authors retain full responsibility for the resulting content.

## Declaration of competing interest

The authors declare no conflicts of interest.
